# CRISPR-Powered Liquid Biopsies in Cancer Diagnostics

**DOI:** 10.3390/cells14191539

**Published:** 2025-10-01

**Authors:** Joshua R. Slattery, Noel Ye Naung, Bernd H. Kalinna, Martin Pal

**Affiliations:** 1 Rural Health Research Institute, Charles Sturt University, Orange, NSW 2800, Australia; jslattery@csu.edu.au (J.R.S.); ynaung@csu.edu.au (N.Y.N.); hkalinna@csu.edu.au (B.H.K.); 2 School of Dentistry and Medical Sciences, Faculty of Science and Health, Charles Sturt University, Wagga Wagga, NSW 2678, Australia; 3 Centre for Dentistry and Oral Health, Charles Sturt University, Orange, NSW 2800, Australia; 4 Gulbali Institute, Charles Sturt University, Wagga Wagga, NSW 2678, Australia

**Keywords:** CRISPR, Cas12, Cas13, cancer, tumour, biomarker, ctDNA, diagnostics, liquid biopsy, point-of-care (POC)

## Abstract

Liquid biopsies promise major advantages for cancer screening and diagnosis. By detecting biomarkers in peripheral blood samples, liquid biopsies reduce the need for invasive techniques and provide important genetic information integral to the emerging molecular classification of cancers. Unfortunately, the concentrations of most biomarkers, particularly circulating tumour nucleic acids, are vanishingly small—beyond the sensitivity and specificity of most assays. Clustered Regularly Interspaced Short Palindromic Repeats diagnostics (herein labelled ‘CRISPR-Dx’) use gene editing tools to detect, rather than modify, nucleic acids with extremely high specificity. These tools are commonly combined with isothermal nucleic acid amplification to also achieve sensitivities comparable to high-performance laboratory-based techniques, such as digital PCR. CRISPR assays, however, are inherently well suited to adaptation for point-of-care (POC) use, and unlike antigen-based POC assays, are significantly easier and faster to develop. In this review, we summarise current CRISPR-Dx platforms and their analytical potential for cancer biomarker discovery, with an emphasis on enhancing early diagnosis, disease monitoring, point-of-care testing, and supporting cancer therapy.

## 1. Introduction

Cancer, as a genetic disease, is driven by mutations in DNA and chromosomal aberrations. These mutations are often found in either tumour-suppressor genes or proto-oncogenes, genes that normally control cell proliferation and cell death [1]. Mutations may be single nucleotide changes, deletions, or insertions, or may involve the insertion of large sequences of foreign viral DNA. Alterations also need not be in the DNA sequence itself. Epigenetic changes, such as changes to DNA methylation, can also result in disruptions to gene regulation. These cancer-driving mutations and alterations lead to uncontrolled cell proliferation, the accumulation of further mutations, and, predictably, the formation of cancers [2].

Cancer is not one disease, but a large heterogeneous group of diseases sharing common mechanisms. Each cancer varies in its overall presentation, progression and response to treatment. As a result, the timely and accurate diagnosis of the location, type, and associated mutations of the cancer is essential for treatment, with significant impact on prognosis [3]. This has become particularly true with the advent of precision medicine, tailoring cancer treatment to the precise mutations present [4]. Traditional histopathological diagnosis, based on architectural and cytological changes, remains the gold standard for most non-haematological malignancies and is integral to accurate diagnosis. Unfortunately, these changes are often not detected until the malignancy has progressed. In 2018, over 50% of lung cancers, and over 40% of colorectal cancers in Australia were classified as late-stage, with regional or distant metastasis at the time of diagnosis [5]. The direct detection of cancer-related biomarkers, including mutations, before morphological changes, therefore, has potential to improve early detection of cancers and improve patient outcomes by facilitating and guiding early treatment.

Particular mutations are strongly associated with poorer health outcomes and treatment resistance in a range of cancers. For example, the gene *TP53*, encoding for tumour suppressor p53, is mutated in 50% of all cancers [6]. Meanwhile, mutations in *erbB2*, coding for receptor tyrosine-protein kinase erbB-2 (HER2), provide a powerful target for cancer therapies [7]. This highlights the molecular classification of cancers as an emerging tool for the guidance of treatment, and as a key principle of precision oncology [4,8]. The adoption of a molecular classification of cancers is particularly prominent in haematological malignancies, once classified by their morphology, now extensively classified by their often-unique mutations and chromosomal aberrations [9]. Haematological malignancies, by their nature, often present with circulating cancer cells, facilitating sampling by relatively non-invasive phlebotomy (blood draw). While circulating tumour cells were first described in 1869 [10], and cell-free nucleic acids in 1948 [11], it was in 1994 that these discoveries facilitated the advent of the liquid biopsy—the detection of cancer-related mutations in DNA circulating in the blood [12]. This creates the potential for a powerful adjunct to traditional diagnostic techniques such as biopsies for solid-tumour cancers, or bone marrow aspirations for haematological malignancies.

The sampling of tumour-derived biomarkers from a relatively non-invasive phlebotomy greatly expands the possibility of screening programmes for early detection, before the presentation of symptoms. This is critical in oncology, with early diagnosis associated with improved survival rates, reduced disease burden, and improved quality of life [13,14]. By detecting mutations associated with specific cancers, liquid biopsies may provide valuable information for prompt diagnosis, early intervention, identification of high-risk patients, and even primary intervention. For example, the *BCR::ABL* fusion gene is the hallmark of chronic myeloid leukaemia, and is reasonably specific to this disease, though it does appear in other leukaemias [15]. While liquid biopsies cannot fully replace traditional biopsies, the detection of specific mutations may help guide further investigations and minimise invasive or expensive procedures such as biopsies and radiographic imaging. These procedures, while essential for staging, grading, and guiding surgical intervention, often come with significant pain or discomfort, and financial cost.

There is, however, a major challenge in the detection of circulating tumour DNA—it is often present at extremely low concentrations and drowned in a sea of non-mutated, nearly identical DNA. Therefore, the techniques used to screen liquid biopsies must be extremely sensitive, and able to differentiate single nucleotide changes. This can present a challenge to the current gold-standard molecular method, quantitative Polymerase Chain Reaction (qPCR). Other technologies, such as digital PCR (dPCR), meanwhile, provide the sensitivity and specificity required, but are expensive, require highly trained personnel, and are entirely laboratory-based [16]. One potential solution is Clustered Regularly Interspaced Short Palindromic Repeats (CRISPR)-based diagnostics, promising not only the sensitivity and specificity needed for these assays, but in many platforms, the potential for point-of-care use.

Point-of-care diagnostics refer to pathology assays performed outside the laboratory setting and typically during or shortly after patient consultation. These assays may take many forms but generally involve handheld or benchtop devices operated by primary care practitioners, such as a nurses or general practitioners. These technologies have grown enormously in their relevance. In oncology, point-of-care diagnostic screening for early-stage cancers—or treatment monitoring—offers the potential to reduce costs and turn-around-times, thereby improving prognosis.

Requiring few reagents and no complex equipment, CRISPR-based assays are well suited to point-of-care use and have been adapted for deployment in lateral flow strips. These simple strips are familiar to both healthcare professionals and the general public as at-home pregnancy tests and rapid antigen tests, familiarised during the COVID-19 pandemic. CRISPR-based lateral flow strips, however, promise specificity and sensitivity vastly greater than rapid antigen tests [17], and potentially greater than laboratory-based qPCR, being comparable to dPCR [18].

This outperformance of antigen-based tests is owed to the innate specificity of the CRISPR associated (Cas) proteins and the sensitivity of the nucleic acid amplification with which they are commonly paired [19]. Additionally, CRISPR-based assays can be relatively simple to re-programme for new targets and testing is greatly accelerated compared to antibody/peptide development and is enhanced by emerging bioinformatic tools that automate this process [20]. CRISPR-based assays offer significant advantages, both in performance and point-of-care suitability. Therefore, this review article summarises the breadth of CRISPR-based diagnostic platforms with an emphasis on their potential to detect specific cancer biomarkers, accelerating disease diagnosis for precision medicine.

## 2. CRISPR—From Gene Editing Tool to Point-of-Care Diagnostics

CRISPR technology has arguably been most famous for its gene-editing applications, since its introduction in 2012 [21]. CRISPR systems are ubiquitous among prokaryotic species and serve as an adaptive immune system against invading nucleic acids (e.g., viral DNA/RNA and transposons), by acting as a genetic repository of known invaders stored within genome of the bacteria, archaea, and even bacteriophages [22]. In prokaryotes, guide RNA, also known as CRISPR RNA (crRNA) is transcribed from the CRISPR locus, along with trans-activating CRISPR RNA (tracrRNA). These RNAs are combined in the presence of the Cas protein and utilised to cleave homologous invading nucleic acids. However, in gene-editing applications crRNA and tracrRNA are synthetically fused to simplify this process. Additionally, many Cas proteins require a protospacer adjacent motif (PAM) either up-stream or down-stream of the target sequence. In nature, the PAM prevents self-cutting of the prokaryote’s own DNA. The PAM, while present in the invading nucleic acid, is not incorporated into the CRISPR locus; therefore the prokaryote’s own DNA is not recognised by the Cas protein [23]. The direction and composition of the PAM is dependent on the Cas protein. For instance, Cas9 requires a PAM of NGG, while Cas12a requires TTTV, where N is an any nucleotide and V is any nucleotide other than thymine [21,24,25]. Several CRISPR systems have been identified and broadly categorised into two classes, composed of six types [26]. Class 1 systems consist of large effector complexes comprising multiple proteins and are further divided into types I, III, and IV. Meanwhile, Class 2 systems utilise a single effector protein and include Cas9 and Cas13, which form types II and VI, respectively. Class 2 also includes type V proteins Cas12, Cas14, and CasΦ/Cas12j [27,28]. A single effector protein makes Class 2 systems significantly easier to work with and have primarily driven the development of CRISPR technology. It is the highly specific binding of Cas proteins to their target sequences, combined with the ease of designing the crRNAs which direct them, that has facilitated the application of this technology in diagnostics. This application has taken the form of many different platforms developed around the various CRISPR-Cas systems and their unique attributes, which are discussed below.

## 3. Overview of CRISPR-Cas Systems Used in Diagnostics

**Figure 1:**
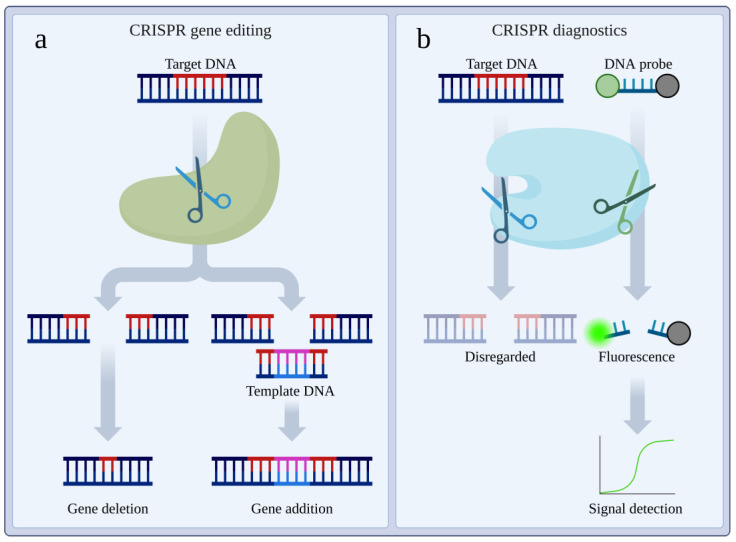
Simplified overview of CRISPR gene editing vs. CRISPR diagnostics. (**a**) CRISPR gene editing is commonly facilitated by the cleavage of target DNA by a Cas protein. The cleaved DNA is then repaired by one of two mechanisms, both utilising the cell’s natural repair processes. Non-homologous end-joining results in the loss or the addition of nucleotides and subsequent gene deletion by frameshift [30]. Homology directed repair utilises a template DNA sequence with flanking regions matching the cleaved target DNA. This template DNA is then incorporated into the genome, resulting in the addition of the gene encoded by the template DNA [30]. (**b**) CRISPR diagnostics commonly utilise Cas proteins with collateral cleavage activity. This activity is activated by the recognition of the target DNA. The collateral cleavage is used to separate a quencher from a fluorophore, resulting in a detectable fluorescent signal. Importantly, this signal is only produced in the presence of the target DNA [38]. Unlike gene editing, in diagnostics, the cleavage of the target DNA is inconsequential, as only the recognition is required.

CRISPR diagnostics (herein referred to as CRISPR-Dx) encompasses a range of platforms utilising Cas proteins for the detection of disease-associated biomarkers. Most commonly, these biomarkers are nucleic acids of pathogens or genetic changes associated with malignancy. These platforms take advantage of the programmable specificity of Cas proteins to detect nucleic acids of interest, utilising a variety of Cas proteins, amplification methods, and result readout strategies (Table 1). In addition to diagnostics, CRISPR technology has also seen development in novel cancer treatments, and extensive use in creation of cancer models [29]. These exciting avenues of research, however, are beyond the scope of this review, which will focus on CRISPR diagnostic systems. Many of these diagnostic platforms, described below, take advantage of the collateral activity of Cas12 or Cas13 to cleave non-specific ssDNA or RNA, respectively, to activate a reporter molecule. By linking the recognition of the target sequence by the Cas protein to a detectable signal, CRISPR-Dx systems leverage the highly specific and sensitive nature of Cas proteins for diagnostics (Figure 1b). This contrasts with the use of Cas9 in gene-editing applications, in which DNA cleavage of the target sequence is the primary goal, after which genetic modification is facilitated commonly by non-homologous end-joining, resulting in gene deletion; or homology directed repair, resulting in gene addition (Figure 1a) [30]. Additionally, in gene-editing applications, collateral off-target activity must be avoided to ensure modification of only the targeted region. CRISPR-Dx platforms, however, benefit from targeting multiple regions within the same genome, allowing an amplification effect, provided that target regions are specific to the tumour or pathogen of interest [20]. Despite the relative ease with which Cas12 and Cas13 have been utilised for CRISPR-Dx systems, the earliest and some of the more recent advances have utilised Cas9. Without the collateral activity exhibited by Cas12 and Cas13, systems utilising Cas9 have employed a variety of novel reporting mechanisms.

### 3.1. Cas9

**Figure 2:**
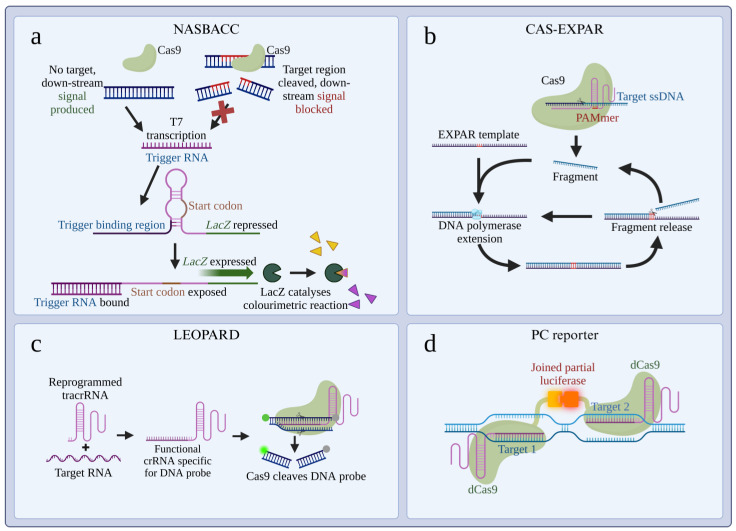
Overview of selected Cas9-based CRISPR-Dx platforms. (**a**) NASBACC utilises Cas9 to cleave a target region of DNA, blocking downstream processing in the toehold switch sensor. In the absence of the target region, T7 transcription converts the DNA to trigger RNA, which subsequently binds the toehold switch sensor. This binding reveals the *LacZ* gene, the product of which catalyses a detectable colourimetric reaction [31]. The preceding DNA amplification and first toehold sensor are not depicted. (**b**) CAS-EXPAR utilises Cas9 to cleave a fragment of target ssDNA. This fragment hybridises to an EXPAR template containing two copies of a region homologous to the fragment. The fragment is extended by DNA polymerase, producing a new copy, which is then liberated by nickase, allowing the process to repeat, producing a detectable amplification via SYBR Green I (not depicted) [32]. (**c**) LEOPARD utilises reprogrammed trans-activating CRISPR RNA (tracrRNA) that combines with target RNA, forming a functional CRISPR RNA (crRNA). This crRNA is specific for a synthetic DNA probe. Cas9 cleaves the DNA probe after fusing with the crRNA, producing a detectable signal [34]. (**d**) PC reporter utilises nuclease deficient Cas9 (dCas9), that lacks nuclease activity. Two crRNAs are used, each specific for adjacent target regions of DNA. When the two dCas9 proteins bind the adjacent regions, attached partial luciferase moieties combine to produce a detectable luminescent signal [35].

CRISPR technology was first leveraged for use in diagnostics utilising Cas9 in the differentiation of Zika virus strains [31]. This system utilises toehold switch sensors to detect viral RNA following isothermal amplification and is therefore termed Nucleic Acid Sequence-Based Amplification CRISPR Cleavage (NASBACC). Cas9 is then used to inhibit the activation of a second toehold sensor based on strain-specific sequences (Figure 2a). Therefore, the system does not directly use Cas9 in the detection of target nucleic acids, and its utilisation of NASBACC limits detection to RNA. Similarly, Cas9 has been combined with Illumina sequencing to detect antimicrobial resistance sequences in a system termed Finding Low Abundance Sequences by Hybridization (FLASH) [33]. These systems do not rely on Cas9 to directly detect pathogens, but to detect specific sequences downstream, informing treatment.

Cas9 has been used to detect pathogens directly, in a method utilising EXponential Amplification Reaction (EXPAR) [32]. This system utilises the recognition and cleavage of target DNA by Cas9 to trigger EXPAR, with the subsequent amplified DNA monitored in real-time using SYBR Green I, and is termed CAS-EXPAR (Figure 2b). This system, interestingly, is the reverse of what would later become the most common strategy in CRISPR-Dx platforms, in which an amplification step precedes the CRISPR identification. One drawback of this approach is that results must be monitored in real time as amplification proceeds regardless of the presence of the target, while in the presence of the target, Cas9 recognition increases the efficiency of the amplification.

CAS-EXPAR and NASBACC were some of the earliest systems, and utilised Cas9’s ability to cleave dsDNA as a primary mechanism in detection. Significant success, however, has been seen in utilising genetically modified Cas9 to directly detect DNA of pathogens. Nuclease-deficient Cas9 (dCas9) is modified from the wild-type to lack endonuclease activity, thereby blocking its ability to cleave its target DNA. dCas9 has been used to directly detect target nucleic acids [35]. In the Paired dCas9 (PC) reporter system, two adjacent regions of the target nucleic acid are targeted with specific crRNAs, each complexed with dCas9. When the two dCas9 proteins bind their targets, attached luciferase moieties on the dCas9 proteins join to produce a luminescent signal, owing to the proximity of the target sequences (Figure 2d). A similar technique has also been utilised for the detection of microRNAs (miRNAs). This technique utilises split horseradish peroxidase (HRP) fragments as the reporter, following rolling circle amplification [36].

In nature, Cas9 utilises two RNA sequences for recognition of the target sequence: a sequence specific crRNA and tracrRNA, which are processed in a complex system, also involving RNase III [46]. In vitro, however, these can be designed as chimeric crRNA:tracrRNA sequences, simplifying the design. Jiao et al. [34] developed a novel approach to CRISPR-Dx, and acronym design, utilising the tracrRNA of Cas9. Leveraging Engineered tracrRNAs and On-target DNAs for PArallel RNA Detection (LEOPARD) utilises a reprogrammed tracrRNA (Rptr) that specifically binds with target RNA forming a functional crRNA. This crRNA then combines with Cas9, allowing it to cleave a specific synthetic DNA reporter (Figure 2c). The major advantage of LEOPARD is that a unique DNA reporter can be designed for each RNA target, permitting significant multiplexing capacity.

### 3.2. Cas13

The identification of Cas13a predates the use of Cas9 as a diagnostic tool [47]. Importantly, the discovery of the collateral cleavage activity of Cas13a greatly simplified CRISPR-Dx approaches [25]. Here, upon binding of Cas13a to its target RNA sequence, collateral activity of the protein breaks down any non-specific adjacent RNAs. By the inclusion of a quenched, fluorescent RNA probe, this can be used as a detection system. This was combined with isothermal amplification via recombinase polymerase amplification (RPA) to create Specific High-Sensitivity Enzymatic Reporter Unlocking (SHERLOCK) [43]. This combination was shown to be more sensitive than RPA with SYBR Green II, or real-time PCR. The addition of RPA also allows detection of DNA targets. Cas13a only binds RNA; however, RPA with T7 RNA polymerase allows for DNA targets to be transcribed into RNA prior to detection by Cas13a, further expanding its application. Cas13 has a significant advantage over other Cas proteins, such as Cas9 and Cas12, in that it does not require the presence of a PAM to recognise its target sequence. Instead, a protospacer flanking site (PFS) is required and varies based on the specific Cas13 used, but is commonly a single non-guanine nucleotide at the 3′ end of the target region [48].

The SHERLOCK system was modified with SHERLOCKv2 to improve sensitivity, enable multiplexing, and enable a visual readout of assay results [45]. Multiplexing was achieved by utilising four subtypes of Cas13, each with differing sequence preferences for collateral RNA cleavage, therefore allowing the use of four distinguishable probes. A visual readout was achieved using a FAM-biotin probe conjugated with RNA and a colloidal gold reporter within lateral flow strips. This change allows CRISPR-Dx to be used without any specialised equipment, providing a significant advantage over laboratory-based platforms.

Further improvements were made to the SHERLOCK system with the introduction of another Sir Arthur Conan Doyle-inspired acronym, Heating Unextracted Diagnostic Samples to Obliterate Nucleases (HUDSON) [44]. The HUDSON system simplifies the extraction of viral nucleic acids and inhibits nucleases present in body fluid samples. It is combined with SHERLOCK for nucleic acid detection and further facilitates the use of SHERLOCK in point-of-care assays by eliminating the need for complex, laboratory-based nucleic acid extraction.

### 3.3. Cas12

Cas12a, previously known as Cpf1, was first confirmed as a single RNA-guided endonuclease in 2015, three years after its first annotation as a CRISPR-associated protein [24]. The simpler mechanism of the protein gave it significant advantages in gene-editing. By processing its own crRNA, Cas12a can be directed by a single crRNA [47]. While initially exploited for multiplex gene-editing [49], the simpler mechanism proved useful in diagnostics.

The trans-cleavage activity of Cas12a was described by two groups in 2018, showing that Cas12a non-specifically cleaves ssDNA, resulting in complete degradation [38,50]. Promisingly, this trans-cleavage only occurred in the presence of a target dsDNA sequence, suggesting that the trans-activity, like that of Cas13, must be activated by the Cas protein binding to its target sequence. This activity was exploited to develop HOLMES (a one-hour, low-cost, multipurpose, highly efficient system), a Cas12a based detection system which utilises a DNA-ligated fluorescent-quencher probe combined with a PCR amplification step [37]. Meanwhile, Chen et al. [38] broke with the literary naming convention with DETECTR (DNA endonuclease-targeted CRISPR trans reporter), which differed from HOLMES by using RPA for target amplification. The use of an isothermal amplification step provides significant workflow advantages, often eliminating the need for expensive, complex equipment. This is reflected in the development of HOLMESv2, which utilises loop-mediated isothermal amplification (LAMP) for target amplification [42]. HOLMESv2 also integrated Cas12b, a thermophilic Cas protein, to allow target amplification by LAMP to occur simultaneously (LAMP operates at approximately 60 °C). Cas12b was also utilised in the CDetection (Cas12b-mediated DNA detection) platform [41]. However, in this case, reactions were performed at 37 °C with RPA used for amplification. The authors report greater sensitivity than those observed with the Cas12a or Cas13 systems.

Chen et al. [38] note that trans-cleavage activity of Cas12 provides an amplification effect owing to its kinetics. That is, following trans-cleavage, the non-specific ssDNA is released from Cas12. This allows Cas12 to catalyse the cleavage of multiple strands of ssDNA, and when paired with an appropriate probe, amplifies the original signal [38]. This mechanism should hold true for any trans-cleavage activity in which the nucleic acid is released after cleavage. Cis-cleavage of the primary target does not generally result in the release of the target nucleic acid from Cas proteins [35].

### 3.4. Cas14

Cas14 is a more recently discovered group of Cas proteins. Compared to other Class 2 Cas proteins (Cas9, Cas12, and Cas13) Cas14 is significantly smaller, at around 40–70 kDa, or approximately half the size of the other proteins [27]. Cas14a targets dsDNA in a PAM-dependent manner and is also able to target ssDNA, but with no PAM or PFS restriction [51]. Much like Cas9, Cas14 requires tracrRNA to process its crRNA in order to bind its target. Unlike the similarly PAM-independent Cas13, the lack of an obligate PFS for target binding or cleavage allows unrestricted freedom in target selection and crRNA design for ssDNA. Cas14a also exhibits non-specific ssDNA cleavage, like that of Cas12a following target binding. These properties facilitated the development of a DNA detection system utilising Cas14a and fluorescent probe, termed Cas14-DETECTR [27].

### 3.5. CasΦ/Cas12j

CRISPR genes have been found to be essentially universal among prokaryote species. The discovery of the ubiquity of huge phages also brought with it the discovery of the ubiquity of CRISPR genes among them [22]. Included in these have been the newly discovered CasΦ, also known as Cas12j, a small type V Cas protein, similar in size and amino acid make up as Cas14 [52]. Also much like Cas14 and Cas12, the protein exhibits non-specific trans-cleavage of ssDNA [28]. While research is limited, the diagnostic potential has been evaluated in combination with exponential amplification reaction (Cas12j) and termed EXP-J. CasΦ targets ssDNA and dsDNA in a PAM dependent manner, and was successfully utilised in the detection of cancer-related miRNAs in clinical samples [28]. CasΦ is the most recently discovered Cas protein in the growing array of Class 2 CRISPR systems, expanding range of tools available for diagnostics (Table 2).

### 3.6. Strategies for Result Readouts

A major advantage of CRISPR-Dx over other technologies, such as dPCR, is its potential for point-of-care use. While early CRISPR-Dx platforms, such as CAS-EXPAR, SHERLOCK, and DETECTR, utilised the measurement of a fluorescent signal, the SHERLOCKv2 platform may have been the first to utilise a point-of-care suitable detection method, using colloidal gold markers in a lateral flow strip format [45]. As a result, as well as due to their ease of use and low expense, lateral flow strips remain a popular method in the development of CRISPR-Dx assays. Others, however, have explored the use of systems to generate an electrical signal as a means of result readout. The E-CRISPR platform utilises a methylene blue electrochemical tag on a ssDNA probe attached to a gold-electrode, such that when the probe is cleaved by Cas12a, the measured current is reduced [39]. This is integrated within a small electronic device also suitable for point-of-care use. A similar approach developed a palm-sized device to detect a fluorescent signal, with the added benefit of including thermal control to allow the use of LAMP as the isothermal amplification method, which operates at approximately 60 °C [53]. These systems, much like the lateral flow strips, are geared towards the potential for point-of-care use, leveraging the major advantage of CRISPR-Dx platforms.

## 4. CRISPR-Dx for Precise and Rapid Cancer Screening

A major challenge in cancer diagnosis and monitoring is the sampling of suspect tumour cells, often necessitating invasive biopsies. Therefore, the possibility of the detecting solid tumour cancer through a relatively non-invasive peripheral liquid biopsy, is at the core of CRISPR-based diagnostics. Similar, more traditional approaches involve the detection of serum tumour markers. These may be proteins or metabolites whose serum levels are changed (often increased) in malignancy [54]. Some of these, such as prostate-specific antigen (PSA), are specific enough to an associated cancer to aid in diagnosis [55]. The detection of elevated PSA, in combination with a physical exam, provides critical information for clinicians, assisting decision-making around more invasive procedures for diagnosis. However, the non-specificity of most tumour markers limits their use to the monitoring of cancer and detection of relapse [55]. Efforts have been made to use tumour markers for screening platforms with little success. Even when multiple tumour markers are screened and combined with machine learning, overall patient benefit, including positive predictive value, remains “inadequate” [56]. Tumour markers are limited primarily by sensitivity, specificity, and biological variation—tumour markers may be elevated in healthy patients, leading to needless invasive procedures, or normal in patients with cancer, resulting in missed diagnoses [54]. For these reasons, their use in screening and diagnostics is limited. As discussed in the introduction, a more recent strategy in liquid biopsies is the detection of cancer-related mutations from circulating tumour DNA.

Circulating cell-free tumour DNA (ctDNA) and miRNAs suggests the possibility of highly specific non-invasive liquid-biopsies [12,16]. However, the concentrations of ctDNA and miRNAs in peripheral blood are extremely low in early disease, often below the sensitivity of gold-standard molecular techniques such as qPCR [16]. This precludes the use of these techniques in the diagnosis of early disease, including screening applications. dPCR offers a system capable of detecting ctDNA at levels as low as 0.01% of all cell-free DNA (cfDNA) [16]. These systems, however, are entirely laboratory-based, requiring highly trained personnel and significant expense. Meanwhile, the more comprehensive assessment allowed by other laboratory-based techniques, such as next-generation sequencing (NGS), has many advantages—namely that investigations are not limited to pre-defined mutations [12]. It is important to note, on the other hand, that the turn-around-times for NGS can vary from days to weeks [57], highlighting the advantage of rapid diagnostics for specific mutations offered by CRISPR-Dx. However, these laboratory-based techniques demonstrate the utility of molecular diagnosis, and have been shown to have clinical validity in the monitoring of breast, lung, and colorectal cancers [58]. This is particularly true for monitoring minimal residual disease—malignant cells remaining after treatment, undetectable by traditional methods. As an example, liquid biopsies have been used to monitor chronic lymphocytic leukaemia for Richter’s transformation, offering greater specificity than traditional methods such as lymphocyte cell counts [59]. However, as identified by Ijzerman et al. [58], the progress of liquid biopsies in early detection lags behind their use in disease monitoring, yet the former may hold significant promise. There is a need for early detection and screening, particularly in a point-of-care format that allows for treatment decisions to be made without lengthy turn-around-times. CRISPR-based diagnostics promise a practical alternative, with true point-of-care capacity, for the detection of these evasive molecular biomarkers.

### 4.1. Biomarkers Enabling CRISPR-Dx Detection

**Figure 3:**
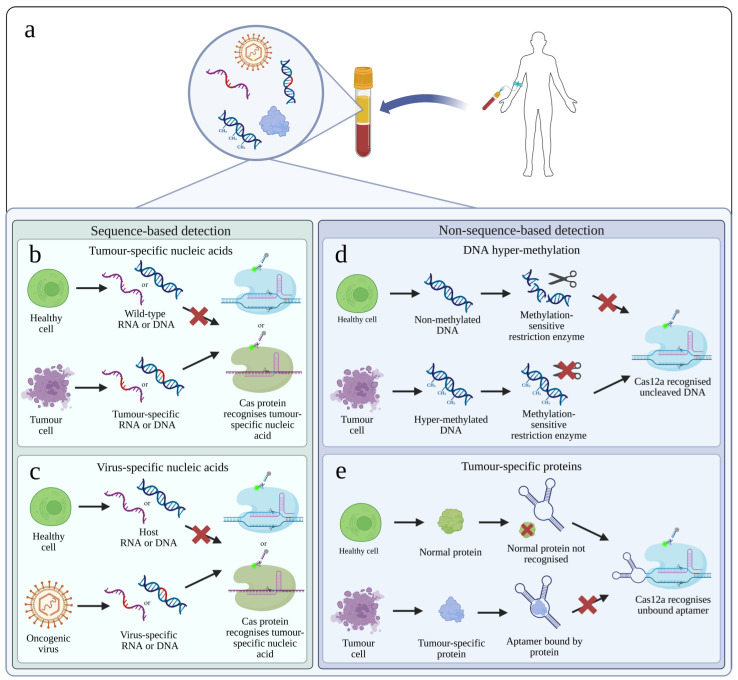
Selected biomarker targets for CRISPR-Dx platforms. (**a**) Liquid biopsies are performed on peripheral blood samples taken by phlebotomy. These samples may contain oncogenic viruses or their genetic material, or tumour-derived DNA, RNA, or proteins. (**b**) Tumour-specific nucleic acids are differentiated by unique mutations that are not present in wild-type nucleic acids from the host. Only the tumour-derived nucleic acids are detected by Cas proteins, owing to the specific crRNA provided [43,60]. (**c**) Similarly, virus-specific nucleic acids contain unique sequences detectable by Cas proteins [61]. (**d**) DNA hyper-methylation is an epigenetic change often seen in tumour DNA. Methylation-sensitive restriction enzymes are used to degrade non-methylated DNA. Thus, Cas12a is able to detect the non-degraded tumour-derived DNA [32]. (**e**) Tumour-specific proteins may be detected by use of DNA aptamers. These aptamers are synthetic three-dimensional DNA structures that strongly and specifically bind target proteins, in this case tumour-specific proteins. Cas12a is programmed via crRNA to detect the aptamer. In the presence of the tumour-derived protein, the aptamers are bound and unavailable for detection by Cas12a, and therefore, no signal is produced [39].

The majority of approaches to cancer diagnosis using CRISPR-Dx utilise liquid biopsies—primarily peripheral blood (Figure 3a). The selection of an appropriate biomarker depends on the nuances of the assay platform and the biological mechanisms that lead to oncogenesis and progression of the cancer in question. Importantly, the clinical specificity of a biomarker (how specific it is for a particular disease) is independent of its analytical specificity (how much other analytes interfere with its detection). As a result, there are many approaches (Figure 3b–e).

#### 4.1.1. ctDNA

The detection of ctDNA was the first cancer biomarker explored in CRISPR-Dx. One of the earliest diagnostic platforms, SHERLOCK, was first utilised in 2017 to detect a range of target nucleic acids, including mock in vitro assays of ctDNA, allowing detection of SNP-containing alleles (Figure 3b) [43]. This mock in vitro assay demonstrated sensitivity in the attomolar range, and the specificity also allowed this detection to take place within a high concentration of unmutated genomic DNA. Gootenberg et al. [43] highlighted that this sensitivity is within the clinically relevant range and is comparable to existing liquid biopsy techniques such as dPCR.

A significant obstacle in the detection of ctDNA remains the overwhelming presence of unmutated or wild-type (WT) DNA within samples. While Cas proteins can exhibit specificity with single-nucleotide resolution, weak signals from minute concentrations of ctDNA can be overwhelmed by non-specific activity targeting WT DNA [60]. EasyCatch is a platform developed to remove both the interfering WT DNA and CRISPR’s, perhaps unhealthy, relationship with acronym-based platform names. This is achieved with restriction enzymes specific to the WT DNA, rendering the WT sequence unable to bind Cas12a. Utilised in the assessment of patient samples, EasyCatch detected ctDNA mutations associated with acute myelocytic leukaemia in under an hour. The use of restriction enzymes also makes this platform highly generalisable.

Detection of ctDNA was the earliest approach in CRISPR-Dx liquid biopsies. Yet, the diminutive concentrations of ctDNA remain a major obstacle. Some researchers have sidestepped the challenge with platforms aimed at the detection of more prevalent biomarkers.

#### 4.1.2. RNA

Similarly to the detection of ctDNA, RNA from tumour cells can be detected in peripheral blood and other specimens (Figure 3b). Detection of RNA has the advantage of detecting not only cancer-specific mutations, but also overexpression of genes, whether mutated or not. Detection of messenger RNA (mRNA) was performed successfully with the HOLMESv2 platform, with the authors noting the potential benefits in tumour progression monitoring [42]. The technique has been applied in the detection of prostate cancer, taking advantage of the overexpression of the long non-coding RNA *Prostate cancer antigen 3* (*PCA3*) [62]. As this was the detection of an overexpression, this platform necessitated the simultaneous detection of a housekeeping gene, *kallikrein related peptidase 3* (*KLK3*). This would be necessary for any determination of overexpression, but requires the platform to be multiplexed, a significant challenge for CRISPR-Dx platforms. The binding of Cas9, rather than cleavage of a reporter, was used to overcome this challenge. Similarly, in many cancers, an overexpressed mRNA is observed, such as in acute promyelocytic leukaemia (APML) in which a fusion of genes—*Promyelocytic leukaemia protein* (*PML*) and *Retinoic acid receptor alpha* (*RARA*) (*PML::RARA*)—is over expressed. Therefore, the detection of the mRNA transcript improves sensitivity over direct detection of the fusion gene in ctDNA, an approach utilised to develop a lateral flow CRISPR-Dx assay for APML [63]. This takes full advantage of the overexpression of the mRNA, which is present with a significantly higher copy number than the ctDNA. Similarly, other promising targets, such as miRNAs, can be greatly overexpressed in certain cancers.

miRNAs are approximately 18–24 nucleotides in length and are extremely stable thanks to their tendency to form hairpin loops. These small non-coding RNAs play an important role in epigenetics by base-pairing with complementary mRNA. miRNAs then destabilise the base-paired mRNA, preventing its translation. miRNAs have been shown to be involved in the development and progression of several cancers, often by post-translationally down regulating the expression of tumour suppressor gene products, or by reduction in regulatory miRNAs that throttle the expression of proto-oncogenes [64]. Therefore, the detection of target miRNAs has significant implications for the diagnosis and management of cancers. Utilising dCas9 and rolling circle amplification (RCA), the down-regulation of miRNAs, specifically let-7a, was detected in the serum of patients with non-small-cell lung cancer (NSCLC) [36]. Let-7a is a known biomarker for NSCLC, demonstrating the feasibility of this approach in a clinical context. Similarly, let-7b is also down-regulated in patient samples, along with other cancer-related miRNAs, using a similar assay that combined Cas13a with RCA [65].

Other groups have also successfully demonstrated the clinical potential of miRNA detection. Clinical samples were evaluated to detect tumour-related miRNA-155 in a method that combines Cas13 with Cas12a [66]. A significant advantage of this dual-Cas method is that no preamplification step is required, which, as the authors note, takes full advantage of the high specificity of the Cas proteins. This system demonstrated a limit of detection (LOD) of 350 aM under ideal conditions, and statistically significant differences between cancer patients and normal controls. However, no LOD was reported for clinical samples. By way of comparison, the implementation of an enzyme-free DNA amplification combined with DNA-based logic gates was able to detect oesophageal-cancer-related miRNA-21 in clinical samples with a LOD of 1.26 fM (a 3.6-fold higher concentration) [67]. It should be noted, however, that the detection of miRNA in this system was performed by an RNA hairpin probe, with Cas12 acting as a final reporter. Therefore, the sensitivity of this system is heavily reliant on the sensitivity of the RNA probe, rather than Cas12.

The detection of ctDNA and various RNAs utilise Cas proteins’ inherent ability to detect nucleic acids in a sequence-specific manner. Some cancer-related genetic changes, however, are not related to mutations in gene sequences. Instead, these are often derived from epigenetic changes such as DNA methylation.

#### 4.1.3. DNA Methylation

DNA methylation is the addition of methyl groups to bases within the DNA, affecting primarily cytosine and adenine. A form of epigenetics, this methylation results in down-regulation of genes, while conversely a lack of methylation results in the up-regulation of genes [2,68]. Tumour cells often display hyper-methylation of tumour suppressor genes or hypo-methylation of proto-oncogenes, contributing to the genetic alterations that result in malignancy. This holds significant value in cancer diagnostics, as DNA methylation has been associated with the development of several cancers [2].

One of the earliest platforms, CAS-EXPAR, experimented with the ability of Cas9 to detect methylation with single nucleotide resolution [32]. The authors noted that the treatment of DNA with sodium bisulphite results in the conversion of cytosine into uracil. This reaction, however, is blocked by methylation of the cytosine. The process, therefore, essentially converts an epigenetic trait (lack of methylation) into a nucleotide change. Cas9 and other Cas proteins can exhibit single nucleotide specificity [69,70]. CAS-EXPAR utilised this resolution, with the conversion of non-methylated cytosine to uracil preventing the recognition of Cas9 at a single nucleotide [32]. It should be noted that the mismatch tolerance of Cas proteins is dependent on the mismatch position and concentration of the target.

Methylation detection has also been achieved by combining Cas12a with the use of methylation sensitive restriction enzymes [71]. In the presence of hyper-methylation, the restriction enzymes are blocked, leaving the target intact. A subsequent reaction allows Cas12a to detect the target and produce a signal. In the absence of methylation, the target is fragmented by the restriction enzymes, such that Cas12a can no longer effectively bind the target, and therefore no signal is produced (Figure 3c).

These approaches expand the diagnostic capacity of CRISPR systems to the detection of epigenetic changes, particularly valuable in the diagnosis and monitoring of cancer. Meanwhile, many potential cancer biomarkers occur far downstream of the genetic alterations, such as the translated proteins. These present challenges for detection with CRISPR-Dx platforms.

#### 4.1.4. Proteins

Proteins are alluring targets as cancer biomarkers, making up most traditional tumour markers, such as PSA and alpha-foetoprotein (AFP). Yet, these proteins present a challenge in CRISPR-Dx as Cas proteins by their nature recognise nucleic acids, not amino acids. This has been overcome by several groups. By using a DNA aptamer, the E-CRISPR platform was able to detect the protein target transforming growth factor ß1, a carcinoma biomarker [39]. The aptamer binds the target protein, with the aptamer concentration thereby reduced. By designing crRNA to target the aptamer, the presence of the protein can be inferred by a reduction in signal from the CRISPR detection system (Figure 3e). By limiting the concentration of the aptamer, quantification of the target protein can be achieved. This was demonstrated and expanded upon by a separate group in the detection of AFP [72]. The approach was refined by the addition of a trigger, taking the form of ssDNA, which binds to the aptamer, but is released when the aptamer binds the protein target. The crRNA is complementary to the trigger, and therefore, the signal produced by Cas12a increases with the concentration of the protein target.

These techniques have been used to great effect by several groups in the detection of protein targets from tumour extracellular vesicles, in a similar manner to the detection of ctDNA. These have included protein biomarkers associated with nasopharyngeal carcinoma and breast cancer [73,74,75,76]. These extracellular vesicles are particularly promising targets as biomarkers, as they circulate readily and contain tumour derived proteins, including transmembrane proteins. The use of aptamers in this context also pairs well with the nucleic acid amplification method hybridisation chain reaction and CRISPR-Dx in the creation of DNA-based logic gates [75,76]. Essentially, this allows the detection of two target proteins, but only when both are present on the membrane of the vesicle, which improves specificity and allows distinction between tumour-derived and host-derived extracellular vesicles. Another option is to use antibodies to trigger a reaction to release a nucleic acid probe detectable by Cas proteins [77]. This is a highly generalisable method; however, it limits specificity to that of the instigating antibody binding, much like traditional antibody-based methods.

The use of aptamers allows CRISPR-Dx platforms to target proteins. However, their use does not capture the activity of enzymes. This is particularly relevant when the activity of the enzyme is directly related to an increased risk of cancer, as is the case for increased levels of flap endonuclease 1 (FEN1). Cas13a was utilised to detect the nuclease activity of FEN1, by instead allowing FEN1 to cleave part of a DNA probe [78]. The cleavage of the 5′ flap in the probe allowed the ligation of the probe and transcription by rolling circle transcription into an RNA sequence recognisable by Cas13a. This, in principle, could be modified to detect the activity of other nucleases; although, it may not be practical or even achievable to generalise this to other classes of enzymes. DNA barcodes, previously utilised by Hao et al. [79] addressed this issue by the design of DNA barcodes attached to a protease substrate. The substrate is specific to the cancer-related protease, such that when it is digested in vivo the DNA barcode is released. A urine sample from the patient is then collected, and the DNA barcode, recognisable by Cas12a, is detected. This method is theoretically generalisable to any protease but does require the DNA barcode to be administered to the patient. As a result, this technique has only been tested in a murine cancer model. This method promises a generalisable, minimally invasive, point-of-care-capable cancer diagnostic tool.

#### 4.1.5. Other Non-Nucleic-Acid Biomarkers

Cas proteins inherently recognise nucleic acids and are well suited to the detection of these molecules. Despite this, many groups have successfully designed CRISPR-Dx platforms capable of detecting a range of non-nucleic-acid targets. This is useful given the range of biomarkers associated with cancers. The previously discussed platforms, however, target specific families of molecules. An attractive goal would be a programable system capable of recognising a broad range of molecular targets. This was achieved, in principle, with utilisation of engineered ribozymes [80]. These RNA enzymes include an RNA aptamer that can be designed to recognise a theoretical arbitrary molecular target. Upon recognition of the target, the ribozyme self cleaves and releases a crRNA that allows Cas12a to recognise a dsDNA substrate and, in turn, cleave a ssDNA probe. This system showed good correlation with high-performance liquid chromatography but had issues with detecting high concentrations of targets in blood filtrate, suggesting some form of interference. While Cas12a was the ultimate reporter, the primary mechanism of this system is the ribozyme recognition, this raises issues present in many of these non-nucleic-acid systems.

Zhao et al. [66] highlight a significant advantage of their dual-Cas12a/Cas13 system—preamplification is not necessary. Preamplification adds complexity to assay design as well as implementation, increasing processing times. The reliance on complex systems to detect non-nucleic-acid targets only compounds the issues. The major strength of CRISPR systems is the high specificity of Cas proteins for their target sequences, and the relative ease with which the guiding crRNA can be designed and manufactured. This advantage is lost when the Cas protein is used as a reporter, activated by a separate target recognition system, as is the case with the use of aptamers, enzymatically cleaved DNA probes, and ribozymes [39,78,80]. In all these systems, the limiting reagent of specificity and sensitivity is the non-Cas detection mechanism, which may fall well short of that exhibited by purely CRISPR-based systems. Ribozyme-based detection, for example, while theoretically capable of detecting any small molecule, was reported with a sensitivity in the nanomolar range [80]. This contrasts with the use of preamplification of nucleic acids, which synergises well with CRISPR-Dx platforms and have reported attomolar detection limits. The limited sensitivity of the Cas proteins is compensated for by the preamplification, while the high specificity of Cas proteins essentially negates issues with non-specific amplification often observed in isothermal amplification methods such as RPA [18].

Many groups have made great advances in novel mechanisms for detecting non-nucleic-acid targets with CRISPR systems. It has been argued, however, that these often-convoluted systems run the risk of failing to capture the high specificity of Cas proteins.

### 4.2. Cancer Associated Viruses

There are many viruses strongly associated with oncogenesis, with approximately 10% of cancers attributable to viral infection [81]. Among these varied viruses, some of the most commonly known include human papillomavirus (HPV), Epstein–Barr virus (EBV), hepatitis B (HBV) and C (HCV) viruses. These viruses utilise a diverse array of mechanisms to trigger oncogenesis. HPV encodes oncoproteins that increase degradation of the critical tumour suppressor p53, while EBV interacts with cellular proteins to upregulate cell proliferation [82]. Conversely, HBV and HCV directly induce mutations by inserting viral DNA into the genome of the host cell, a mechanism compounded by the chronic inflammation of hepatitis, which is well associated with oncogenesis [83]. These mechanisms often compound, creating a strong correlation between infection and cancer risk. In some cases, such as cervical cancer, correlation has been shown to be strong enough that screening methods have moved away from direct detection of malignant cells or tumours, and towards detection of viral infection (in this case HPV), with the successful implementation of an Australian national screening programme [84].

Viral nucleic acids are present in human samples at much higher concentrations than ctDNA and naturally form an attractive target for CRISPR-Dx. The DETECTR platform was trialled early on to detect HPV DNA in crude patient samples, detecting HPV in 48 samples out of 50 detected with PCR [38]. This assay was performed at 37 °C, using RPA for nucleic acid amplification. RPA, as opposed to PCR, requires essentially no equipment, though detection via fluorescent signal still restricted this early assay to the laboratory. The E-CRISPR platform was also tested for its ability to detect HPV16 DNA in human serum [39]. E-CRISPR is a hand-held point-of-care device facilitated by omitting an amplification step, though this reduces sensitivity, finding a detection limit of 50 pm.

Point-of-care capability with high sensitivity was achieved with Cas12a utilised in combination with HUDSON for DNA extraction and RPA for amplification to detect HPV16 and HPV18 DNA in plasma from patients with known HPV positive cervical cancer [61]. Importantly, these results were visualised with lateral flow strips, requiring little technical skill and no complex equipment. The study also determined the limit of detection for their assay, finding it to be 240 aM and 280 aM for HPV16 and HPV18, respectively. Similarly, also in 2019, the SHERLOCK platform was modified by Wu et al. [85] to detect EBV DNA and to be performed at room temperature. This has significant advantages over the previous platforms using RPA which were performed at 37 °C, increasing its practicality and applicability to point-of-care use. Other researchers have also focused on EBV, particularly in the context of screening for nasopharyngeal carcinoma risk, and have greatly improved upon the sensitivity of other CRISPR-Dx assays [86]. This study developed an amplification-free digital-droplet-based assay that requires no nucleic acid amplification yet exceeded previous sensitivities. The system detected just five copies of target DNA per µL, confirmed by digital-droplet PCR (ddPCR). EBV is a group 1 carcinogen, associated with non-Hodgkin’s lymphoma, Hodgkin’s lymphoma, Burkitt’s lymphoma, as well as oral and oropharyngeal complications [87]. Importantly, EBV status may provide important prognostic information to clinicians, highlighting the value of a point-of-care assay [88].

Significant progress has been made in the development of CRISPR-Dx assays to detect cancer-related viruses. While the detection of targets such as ctDNA shows promise, the detection of viral nucleic acids may be closer to clinical implementation [89]. Promisingly, this is not limited to viruses—other known cancer-related pathogens include (but are not limited to) bacteria, such as *Helicobacter pylori,* which is known to cause gastric cancer [90], and parasites, such as *Schistosoma haematobium* which is known to cause bladder cancer [91]. There is potential to broaden the scope of CRISPR-Dx in cancer diagnosis to include the detection of a wide range of pathogens as well as the detection of ctDNA.

## 5. Comparison of Performance Among CRISPR-Dx Platforms

### 5.1. Biomarker Capacity

A broad range of CRISPR-Dx platforms has been developed, employing diverse Cas proteins, detection methods, and amplification techniques (Table 1), with analytical performance varying substantially across platforms (Table 3). As previously discussed, there are many potential biomarkers for cancer detection. Several CRISPR-Dx platforms have explored these diverse options, leveraging CRISPR-Dx to detect non-nucleic-acid targets. E-CRISPR is a notable example, employing DNA aptamers to detect target proteins with an LOD of 0.2 nM—a novel strength of the platform [39]. While novel, it should be noted that such techniques are not inherent to particular platforms—aptamers could be theoretically integrated into almost any CRISPR-Dx platform. Continuing in this vein, many platforms were initially demonstrated on either DNA or RNA; however, the use of RNA polymerase or reverse-transcriptase allows Cas proteins specific for one nucleic acid to detect the other, albeit with some added complexity.

### 5.2. POC Capacity: Trade-Offs in LOD and Time-to-Result

The E-CRISPR platform also boasts significant POC capacity with a custom-built electronic device for assay readout. This contrasts with many other platforms in the literature, which often describe POC potential but lack practical implementation. However, a major drawback of E-CRISPR is its significantly higher LOD for nucleic acid targets—50 pM, six orders of magnitude greater than the ~1 aM reported by most other platforms. This primarily reflects E-CRISPR’s omission of an amplification step, which also allows the platform to have one of the shortest time-to-result values (30 min), at the cost of sensitivity. This trade-off is an important factor to consider. Time-to-result varies considerably among platforms, ranging from 23 min to 5 h. By way of comparison, POC qPCR instruments deployed in remote settings offer a time-to-result of 60–90 min. Several CRISPR-Dx platforms outperform this value, with more recent platforms also maintaining high sensitivity.

An attomolar LOD is the common sensitivity target for most platforms. However, Gootenbergnoted that some applications—such as HIV detection—require sub-attomolar detection ranges [45]. In their SHERLOCKv2 platform, they utilised an extended RPA step, which prolonged the time-to-result but reduced the LOD to 8 zM—approximately 1000-fold lower than 1 aM. This represents the lowest reported LOD for any platform examined. This extremely low LOD surpasses that of qPCR, which Forootan et al. estimated to be 3 molecules in a reaction volume of 1.6 µL—equating to <10 aM under ideal conditions [92]. Notably, SHERLOCKv2 has been developed with an option for POC lateral flow readout. In fact, most CRISPR-Dx platforms meet or exceed the <10 aM benchmark, while also offering capacity—or at minimum, potential—for POC application beyond that of most qPCR platforms. However, it should be noted that laboratory-based technologies, such as dPCR, generally provide the LOD at around 0.5 target copies per µL (~0.8 zM) [93].

In addition to LOD, analytical performance is defined by specificity—the ability to discriminate the target analyte from non-targets. All CRISPR-Dx platforms examined, except PC reporter, achieved single-nucleotide resolution (Table 3). However, most did not quantify analytical specificity, instead demonstrating no cross-reactivity with closely related off-target samples or sequences. While this absence of cross-reactivity could be interpreted as 100% specificity, such conclusions are limited by the relatively small sample sizes used. Larger, more diverse validation studies are needed to ascertain true specificity values. Of the platforms examined, only FLASH, HUDSON-SHERLOCK, and EXP-J reported a quantified analytical specificity, at 99.9%, 100%, and 95%, respectively [28,33,44]. This makes comparisons of specificity performance between platforms or with other technologies difficult.

An additional major factor in POC capacity is cost. Among platforms reporting per-assay costs, estimates were almost universally below $1.00 USD per assay. Most of these platforms also require no specialised equipment, further reducing costs. As a comparative benchmark, tuberculosis detection using the widely deployed POC qPCR analyser, GeneXpert, was priced at $7.97–14.90 USD per assay in 2023 (sold at cost of manufacture), with the benchtop instrument itself costing $17,500–19,000 USD [94].

### 5.3. Clinical Sensitivity and Specificity

LOD is a measure of analytical sensitivity, but clinical sensitivity—an assay’s ability to detect known positive patient samples (i.e., 1 minus the false-negative rate)—is equally important in assay evaluation. Among the platforms reviewed, only DETECTR and Wax-CRISPR reported clinical sensitivity. DETECTR tested 50 known HPV-positive patient samples, with qPCR as the benchmark, and achieved a 96% detection rate [38]. Wax-CRISPR was evaluated on 100 patient samples for its ability to detect six pathogens of the female genital tract, including HPV 16 and 18, achieving 98.4% clinical sensitivity [40].

As with clinical sensitivity, clinical specificity differs from its analytical counterpart. Clinical specificity denotes an assay’s ability to correctly identify true-negative samples (i.e., 1 minus the false-positive rate). Amongst the reviewed platforms, only Wax-CRISPR reported clinical specificity—94.7%, equating to a false-positive rate of 5.3%. [40]. Additionally, an application of the DETECTR platform by an independent group demonstrated a clinical specificity of 100% when screening patient samples for APML [63].

False positives may arise from off-target binding of the Cas protein. Such effects in CRISPR technologies are mitigated though careful crRNA design, as well as variant Cas proteins [89]. Another possibility is sample contamination, which can be an issue if aerosol generation is not carefully controlled [53].

Unfortunately, the overall lack of clinical metrics—such as false-positive and false-negative rates—remains a significant limitation in assessing the translational capacity of CRISPR-Dx.

### 5.4. ctDNA Sensitivity: Measure of Variant Allelic Fraction

The LOD for ctDNA is most often described in terms of variant allelic fraction. That is, the lowest fraction of total cell-free DNA (cfDNA) that consists of ctDNA that is reliably detected. In patients with cancer, variant allelic fractions can vary dramatically—from over 90% down to 0.01%, with levels rarely observed below this threshold [12,45]. qPCR typically detects variant allelic fractions down to 10%, whereas dPCR and NGS typically achieve sensitivities of 0.01% and ≤0.04%, respectively [95]. Few CRISPR-Dx platforms report the LOD for allelic fraction. Among those examined, SHERLOCK and SHERLOCKv2 report the lowest LODs of 0.1% and 0.6%, respectively [43,45]. However, these figures are based on only four samples per assay. The higher LOD reported for SHERLOCKv2 reflects its use of clinical patient samples, where 0.6% was the lowest variant allelic fraction present. In contrast, the older platform SHERLOCK was validated using mock ctDNA samples spiked with mutations, with 0.1% being the lowest fraction tested. Therefore, it remains unknown whether the platforms can detect variant allelic fractions below these thresholds. Importantly, as discussed, variant allelic fractions in patients may be as low as 0.01% in early-stage cancers or in malignancies with inherently low ctDNA levels [12,58]. Currently, only dPCR and NGS are known to be capable of detecting fractions this low [95]. As a result, the true analytical sensitivity of CRISPR-Dx for ctDNA detection remains uncertain, posing challenges for establishing its potential clinical utility.

**Table 3:** Comparison of CRISPR-Dx platforms. Tested biomarkers include those examined in the original article describing the platform.

CRISPR-Dx Platform	Tested Biomarkers	Limit of Detection	Time-to-Result	Point-of-Care Capacity
NASBACC [31]	RNA	≤10 fM	>60 min	✔
CAS-EXPAR [32]	DNA	≤10 aM	≤60 min	✔
CAS-EXPAR [32]	DNA methylation	≤10 aM	–	–
FLASH [33]	DNA	≤10 aM	–	–
LEOPARD [34]	RNA	≤10 aM	–	✔
PC reporter [35]	DNA	>10 fM	>60 min	✔
RCA-CRISPR-split-HRP [36]	mRNA	≤10 fM	>60 min	✔
HOLMES [37]	DNA/RNA	≤10 aM	≤60 min	–
DETECTR [38]	DNA	≤10 aM	≤60 min	✔
E-CRISPR [39]	DNA	>10 fM	≤30 min	✔
E-CRISPR [39]	Protein	>10 fM	>60 min	✔
Wax-CRISPR [40]	DNA	≤10 aM	≤30 min	✔
CDetection [41]	DNA	≤10 aM	≤30 min	✔
CDetection [41]	ctDNA	1% VAF ^1^	≤30 min	✔
HOLMESv2 [42]	DNA/RNA	≤10 aM	≤30 min	✔
HOLMESv2 [42]	DNA methylation	–	–	–
SHERLOCK [43]	RNA	≤10 aM	≤60 min	✔
SHERLOCK [43]	DNA	≤10 aM	>60 min	✔
SHERLOCK [43]	ctDNA	0.1% VAF ^1^	>60 min	✔
HUDSON-SHERLOCK [44]	RNA	≤10 aM	>60 min	✔
SHERLOCKv2 [45]	RNA/DNA	≤10 aM	≤30 min	✔
SHERLOCKv2 [45]	ctDNA	0.6% VAF ^1^	≤30 min	✔
Cas14-DETECTR [27]	DNA	–	>60 min	–
EXP-J [28]	mRNA	≤10 fM	≤60 min	–

^1^ Variant allelic fraction. Checkmark (✔) indicates POC readiness.

## 6. Challenges and Future Directions in CRISPR-Dx for Cancer Diagnosis

### 6.1. Challenges in Detecting Elusive ctDNA

Liquid biopsies have promised to supplement traditional cancer diagnostic techniques, provide earlier diagnoses, and enhance disease monitoring. The clinical application of liquid biopsies, however, remains elusive. Several obstacles hinder development, including technical challenges in the detection of vanishingly low concentrations of cancer-derived biomarkers. CRISPR-based diagnostic platforms have the potential to alleviate this issue, with the additional major advantage of point-of-care capacity.

Some of the earliest work focused on the potential for detection of ctDNA—perhaps the most direct strategy in cancer cell detection, but also the most technically challenging in terms of biomarker concentration in peripheral blood. Despite this early work, this area of research is the least explored, with relatively few newer studies focusing on the detection ctDNA. The more recent studies have instead often found success in the detection of other nucleic acids, such as mRNAs, miRNAs, and long non-coding RNAs. Similarly, the detection of cancer-related viruses has shown promise, while others have expanded the platform to the detection of non-nucleic-acid targets. These strategies exploit the relatively high abundance of these biomarkers compared to ctDNA, providing an easier target for detection.

Although successful, this highlights that the technology requires further refinement in the direct detection of ctDNA. Importantly, many cancers, such as leukaemias, require the identification of specific genetic mutations for a definitive diagnosis [9]. Therefore, the detection remains largely limited to screening and monitoring purposes. IJzerman et al. [58] show that ctDNA detection platforms, regardless of technology, lag most in diagnostic applications, while surveillance and treatment selection are far closer to clinical application. Nevertheless, these ctDNA detection platforms have demonstrated both technical and clinical validity.

This is mirrored in CRISPR-Dx, in which early platforms such as SHERLOCK have been used to detect ctDNA in clinical samples [45]. These platforms are powerful tools, and many studies have focused on the development and advancement of their applications. However, as highlighted by Wang et al. [89], there is a need for clinical trials to determine the clinical validity and utility of CRISPR diagnostic platforms, particularly in the diagnosis and monitoring of cancers. The development of these platforms will provide a powerful tool to clinicians.

### 6.2. Point-of-Care Deployment Challenges

Other technologies, such dPCR, provide extreme sensitivities and absolute quantification. While some point-of-care systems are in development, they remain primarily laboratory-based approaches [95,96]. In contrast, many CRISPR-Dx platforms have been designed with point-of-care use in mind. This may well be where the true utility of CRISPR-Dx lies.

Laboratory medicine plays an integral role in diagnostics, and CRISPR-Dx will not replace existing or emerging laboratory-based technologies. However, trends of laboratory consolidation and centralisation risk exacerbating issues with delayed diagnoses. This is particularly relevant among regional and remote communities, who already suffer from poorer health outcomes compared to their urban counterparts [97].

The development of point-of-care based diagnostics has the potential to complement, rather than replace, centralised laboratories by filling in the diagnostic gaps [98]. This is possible by reducing turn-around-times of time-critical diagnoses and allowing clinicians to diagnose and treat (or refer) a patient in a single visit. As mentioned, CRISPR-Dx has shown a great deal of promise in point-of-care use. This is primarily facilitated by the highly sensitive nature of the technology, combined with minimal equipment dependencies.

Early research in CRISPR-Dx quickly focused on point-of-care capacity, with the development of SHERLOCKv2 introducing a lateral flow strip readout [45]. This has paired well with existing molecular technologies, such as isothermal nucleic acid amplification (e.g., LAMP and RPA). This has clear benefits in the diagnosis of diseases such as infections, which are often suitable for diagnosis and treatment in a single visit.

For cancer, the major utility may instead lie in disease monitoring. As discussed in the context of ctDNA detection, the detection of minimal residual disease has implications in the monitoring of cancer post-treatment. Certain cancers, however, do require rapid diagnosis. APML is infamous for its tendency to induce disseminated intravascular coagulopathy, leading to rapid death. Without treatment, the median survival is approximately one month, while with treatment, the two-year survival rate is 97%—exemplifying the importance of rapid diagnosis [99].

For these reasons a Cas12a diagnostic method utilising LAMP was developed to diagnose APML in three hours, with minimal equipment and training [63]. This method highlights the benefits of not only point-of-care, but ‘near-point-of-care’ approaches to diagnostics—that is, simplified, cost-effective methods suitable for use in regional and peripheral laboratories and hospitals. In this example of APML, this removes delays caused by specimen referral to centralised laboratories, and alleviates issues stemming from reliance on older techniques like microscopy.

The practical integration of CRISPR-Dx into hospital and laboratory workflows requires consideration. However, this process is likely simpler than that of other technologies, primarily due to the limited equipment and training required. Among the described CRISPR-Dx platforms developed for POC use, two strategies are most commonly employed: simple benchtop or handheld electronic devices, and lateral flow strips. Antigen-based lateral flow strips are already used in hospitals and laboratories—for example, in the detection of SARS-CoV-2 and malarial parasites [100]. In contrast, the electronic devices present greater challenges for integration. Laboratory and hospital staff will require training in their use, as these devices are commonly custom built. However, none of these devices have been commercialised. While a commercialised device would likely be standardised and adhere to industry norms of operation, this underscores another challenge facing CRISPR-Dx.

### 6.3. Clinical and Regulatory Challenges

Currently, the commercialisation of CRISPR-Dx remains in its infancy, with few applications having received regulatory approval. Mammoth Biosciences and Sherlock Biosciences both had CRISPR-based SARS-CoV-2 assays approved in the United States under the Emergency Use Authorization (EUA) authority in 2022 [101,102,103]. These approvals restrict the assays to laboratories certified for complex testing. EUA approval is also temporary—Mammoth Biosciences’ authorisation has since been revoked. The regulatory approval of POC CRISPR-Dx faces additional challenges, particularly in the simplification and standardisation of protocols. These protocols vary considerably between research groups and currently may be too complex for clinical or at-home use [101]. Additionally, the storage and transport of reagents present challenges, as they often require constant refrigeration and are less amenable to POC deployment. However, lyophilisation of reagents has been shown to enable room-temperature storage of CRISPR and RPA reagents, with no appreciable impact on performance [104].

There is a significant gap in the clinical validation of CRISPR-Dx—a challenge common to many liquid biopsy technologies [58]. The majority of CRISPR-Dx platforms described above have been preliminarily validated on synthetic samples, mock-patient specimens, or small patient cohorts. Larger-scale clinical trials are needed to validate the clinical efficacy of CRISPR-Dx, particularly in liquid biopsy applications. As discussed previously, there is limited data on the ability of CRISPR-Dx platforms to detect low variant allelic fractions, underscoring a need to assess technical development and clinical validation in liquid biopsies using CRISPR-Dx platforms.

### 6.4. Future Directions for CRISPR-Dx

CRISPR-Dx platforms invariably integrate complementary technologies to enhance assay performance. Most commonly, nucleic acid amplification is used to improve sensitivity. Meanwhile, custom electronic devices enhance portability and enable POC applications. Ibrahim et al. propose the integration of artificial intelligence (AI) with CRISPR-Dx platforms [105]. Their biosensor would employ a collection of Cas enzymes—Cas9, Cas12a, and Cas13a—to detect biomarkers. The output from the CRISPR-based detection would be analysed via AI-driven cloud computing to provide clinically relevant insights. However, the specific biomarkers that would be targeted by the system are not disclosed.

A significant limitation in CRISPR-Dx is the difficulty in developing multiplexed assays. The challenge arises from the non-specific nature of collateral cleavage, which is most commonly used to generate an output signal. This limitation was addressed by the SHERLOCKv2 and LEOPARD platforms, as described earlier. More recently, a novel approach has utilised AI integration to overcome this constraint. This platform employs an AI model to analyse the visual output of a microfluidic chip and predict the detected pathogen [106]. The output consists of a series of bubbles produced by a CRISPR-based or antigen-based hydrogen peroxide reaction, enabling detection of HBV, HCV, HIV, SARS-CoV-2, and Zika virus. While human interpretation is unreliable, the integration of AI enabled a bubble pattern across the microfluidic chip that is unique to each virus, thereby achieving 100% sensitivity and specificity in detection of SARS-CoV-2.

CRISPR-Dx may also be integrated with other emerging technologies, such as nanotechnology and wearable devices. While no such platforms are currently described, AI-integrated, wearable CRISPR-Dx platforms could theoretically monitor patients for health-related biomarkers in real time and provide critical information to both clinicians and patients [107]. Advances in aptamer and nanoparticle technology are likely to facilitate the use of CRISPR-Dx in detecting essentially arbitrary biomarkers [108]. This has already been demonstrated in principle by several platforms, notably E-CRISPR [39]. Integration of these technologies with high levels of multiplexing may enable continuous monitoring of key biomarkers, while AI tools could facilitate the processing of the large volumes of data to support accurate and impactful health decisions.

Nearly a decade has passed since CRISPR-based diagnostics were first investigated. In that time, several new Cas proteins have been discovered and leveraged to detect a plethora of nucleic acid and non-nucleic-acid targets. In the coming years, CRISPR-Dx will likely become a powerful tool available to clinicians in the diagnosis of cancer.

## Figures and Tables

**Table 1:** Summary of selected CRISPR-Dx platforms.

CRISPR-Dx Platform	Year	Cas	Primary Target ^1^	NAA ^2^	Multiplexing	Readout
NASBACC [31]	2016	Cas9	RNA	NASBA ^3^	No	Colourimetric
CAS-EXPAR [32]	2018	Cas9	ssDNA	EXPAR ^4^	No	Fluorescence
FLASH [33]	2019	Cas9	dsDNA	PCR ^5^	Yes	Illumina sequencing
LEOPARD [34]	2021	Cas9	RNA	PCR	Yes	Gel Electrophoresis
PC reporter [35]	2017	dCas9	dsDNA	PCR	No	Luminescence
RCA-CRISPR-split-HRP [36]	2018	dCas9	miRNA	RCA ^6^	No	Colourimetric
HOLMES [37]	2018	Cas12a	DNA	PCR	No	Fluorescence
DETECTR [38]	2018	Cas12a	DNA	RPA ^7^	No	Fluorescence
E-CRISPR [39]	2019	Cas12a	dsDNA	None	No	Electrical
Wax-CRISPR [40]	2025	Cas12a	dsDNA	RPA	Yes	Fluorescence
CDetection [41]	2019	Cas12b	DNA	RPA	No	Fluorescence
HOLMESv2 [42]	2019	Cas12b	DNA	LAMP ^8^	No	Fluorescence
SHERLOCK [43]	2017	Cas13a	RNA	PCR	No	Fluorescence
HUDSON-SHERLOCK [44]	2018	Cas13a	RNA	RPA	No	Lateral flow strip
SHERLOCKv2 [45]	2018	Cas13a Cas13b Cas12a	RNA	RPA	Limited	Lateral flow strip
Cas14-DETECTR [27]	2018	Cas14a	ssDNA	PCR	No	Fluorescence
EXP-J [28]	2024	CasΦ	DNA	EXPAR	No	Fluorescence

^1^ Many platforms optionally utilise RNA polymerase or reverse-transcriptase to target nucleic acids other than the primary target, though this adds complexity. Only primary targets are shown. ^2^ NAA = nucleic acid amplification. ^3^ NASBA = nucleic acid sequence-based amplification. ^4^ EXPAR = exponential amplification reaction. ^5^ PCR = polymerase chain reaction. ^6^ RCA = rolling circle amplification. ^7^ RPA = recombinase polymerase amplification. ^8^ LAMP = loop-mediated isothermal amplification.

**Table 2:** Cas proteins commonly used in CRISPR-Dx platforms.

Cas Protein	Cas9	Cas12	Cas13	Cas14	CasΦ
**Figure**	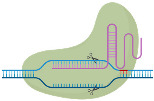	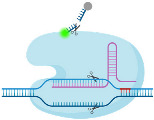	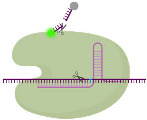	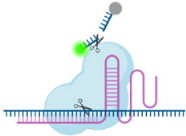	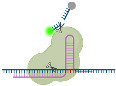
**Example platform**	LEOPARD [34]	DETECTR [38]	SHERLOCK [43]	Cas14- DETECTR [27]	EXP-J [28]
**Target**	dsDNA	dsDNA	RNA	ss/dsDNA	ss/dsDNA
**tracrRNA required**	Yes	No	No	Yes	No
**PAM ^3^** **/PFS ^4^** **restricted**	PAM (NGG) ^1^	PAM (TTTV) ^1^	PFS (H) ^1^	None/PAM (TTTA) ^1,2^	PAM (AAA) ^1^
**Trans-cleavage**	No	ssDNA	RNA	ssDNA	ssDNA

^1^ A = adenine; G = guanine; T = thymine; N = any nucleotide; V = any nucleotide except thymine; H = any nucleotide except guanine. ^2^ Cas14 is only PAM-restricted when targeting dsDNA. ^3^ PAM = Protospacer adjacent motif. ^4^ PFS = Protospacer flanking site.

## Data Availability

No new data were created in this study.

## Abbreviations

The following abbreviations are used in this manuscript:

AI	Artificial intelligence
AFP	Alpha-foetoprotein
APML	Acute promyelocytic leukaemia
Cas	CRISPR associated
CAS-EXPAR	CRISPR associated EXPAR
CDetection	Cas12b-mediated DNA detection
cfDNA	Cell-free DNA
COVID-19	Coronavirus disease 2019
CRISPR	Clustered regularly interspaced palindromic repeats
CRISPR-Dx	CRISPR diagnostics
crRNA	CRISPR RNA
dCas9	Nuclease-deficient Cas9
ddPCR	Digital droplet PCR
DETECTR	DNA endonuclease-targeted CRISPR trans reporter
DNA	Deoxyribonucleic acid
dPCR	Digital polymerase chain reaction
dPCR	Digital PCR
dsDNA	Double-stranded DNA
EBV	Epstein–Barr virus
EUA	Emergency Use Authorization
EXPAR	Exponential amplification reaction
EXP-J	Exponential amplification reaction (Cas12j)
FEN1	Flap endonuclease 1
FLASH	Finding low abundance sequences by hybridization
HBV	Hepatitis B virus
HCV	Hepatitis C virus
HER2	Tyrosine-protein kinase erbB-2
HOLMES	A one-hour low-cost multipurpose highly efficient system
HPV	Human papilloma virus
HRP	horseradish peroxidase
HUDSON	Heating unextracted diagnostic samples to obliterate nucleases
*KLK3*	*Kallikrein related peptidase 3*
LAMP	Loop-mediated isothermal amplification
LEOPARD	Leveraging engineered tracrRNAs and on-target DNAs for parallel RNA detection
LOD	Limit of detection
miRNA	Micro RNA
mRNA	Messenger RNA
NAA	Nucleic acid amplification
NASBACC	Nucleic acid sequence-based amplification CRISPR
NGS	Next-generation sequencing
NSCLC	Non-small-cell lung cancer
PAM	Protospacer adjacent motif
PC	Paired dCas9
*PCA3*	*Prostate cancer antigen 3*
PCR	Polymerase chain reaction
PFS	Protospacer flanking site
PSA	Prostate-specific antigen
qPCR	Quantitative polymerase chain reaction
RAT	Rapid antigen test
RCA	Rolling circle amplification
RNA	Ribonucleic acid
RPA	Recombinase polymerase amplification
Rptr	Reprogrammed tracrRNA
SARS-CoV-2	Severe acute respiratory syndrome coronavirus 2
SHERLOCK	Specific high-sensitivity enzymatic reporter unlocking
ssDNA	Single-stranded DNA
tracrRNA	Trans-activating CRISPR RNA
WT	Wild-type
